# A Unique Comparison of Tooth Extraction Before and After Emicizumab Prophylaxis in a Patient With Haemophilia A and Inhibitors

**DOI:** 10.1155/crid/8128415

**Published:** 2025-05-22

**Authors:** Miki Zaizen, Takahiro Yagyuu, Sachiko Yata, Hiroshi Nakamura, Mitsuhiko Imada, Nobuhiro Yamakawa

**Affiliations:** Department of Oral and Maxillofacial Surgery, Nara Medical University, Kashihara, Nara, Japan

**Keywords:** emicizumab, haemophilia A, medical cost, postextraction bleeding

## Abstract

Haemophilia A is an inherited X-linked bleeding disorder caused by Factor VIII deficiency; approximately 30% of the patients with haemophilia A develop inhibitors against Factor VIII. Emicizumab has been licenced for the prevention of bleeding in patients with haemophilia A with inhibitors and has demonstrated an 87% reduction in the annualised bleeding rate compared with on-demand therapy in patients with haemophilia A with inhibitors. Emicizumab is approved not only for patients with inhibitors but also for those without inhibitors. However, no reports exist on intraindividual comparisons of perioperative management of tooth extraction before and after emicizumab prophylaxis. This case report describes the perioperative management of similar tooth extractions in the same patient with haemophilia A with inhibitors before and after the initiation of emicizumab. This report provides a unique opportunity for intraindividual comparison of the usage of bypassing agents, postextraction bleeding, and medical costs with and without emicizumab. Furthermore, our report also supports the hypothesis that emicizumab is superior in preventing postoperative bleeding complications.

## 1. Introduction

Haemophilia A, an inherited X-linked bleeding disorder caused by factor (F) VIII deficiency, affects one in 5000–10,000 male births [1]. Approximately 30% of patients with haemophilia A (PwHAs) develop inhibitors against the exogenous FVIII [2]. Emicizumab (Hemlibra), a bispecific monoclonal antibody, has been licenced to suppress a bleeding tendency in PwHAs and patients with haemophilia A with inhibitors (PwHAs-I) since 2017 [3–7]. However, the reported experience on dental surgeries in PwHAs-I on emicizumab is limited [4, 8, 9]. Herein, we report a unique comparison of tooth extraction before and after the initiation of emicizumab prophylaxis in the same PwHA-I.

## 2. Case Report

A 53-year-old man with severe haemophilia A and high-titre inhibitor (historical peak titre: 138 Bethesda units) was referred to our department for the extraction of Tooth 35 with severe periodontitis in 2018. He previously underwent a similar extraction for Teeth 15 and 16 with periodontitis in 2011 and received on-demand therapy for acute bleeding episodes at that time. On the day of the operation under hospitalization in 2011, he received an 80 U/kg bolus of activated prothrombin complex concentrate (aPCC) 1 h prior to extraction. Teeth 15 and 16 were extracted using forceps under infiltration anaesthesia. A local haemostatic method was employed based on our institutional protocol, which includes a socket filled with a gelatin sponge and placement of a custom-made mouth splint [10–14]. He received an aPCC 80 U/kg bolus every 12 h on Postoperative Days (PODs) 0–3. Owing to postextraction bleeding on the first 3 days, aPCC administration was discontinued; he received 270 *μ*g/kg recombinant activated FVII (rFVIIa) boluses every 24 h on PODs 4–7. No bleeding was observed after POD 4; he was discharged on POD 7 (Table 1). No anaemia or thrombotic complications were observed.

In 2013, emicizumab prophylaxis was initiated as part of a clinical trial, post which his first surgical procedure was performed in 2018, involving an extraction of Tooth 35 with periodontitis. On the operation day, he received 90 *μ*g/kg rFVIIa 1 h prior to extraction performed using forceps. Local haemostasis was performed similar to that in 2011. No additional bolus of rFVIIa was administered. He was discharged on POD 3. No bleeding or thrombotic complications were observed. In 2019 and 2021, he underwent extraction of Teeth 36 and 17, respectively, similar to that in 2018. No postoperative rFVIIa infusion was administered in 2019, while an additional bolus of 90 *μ*g/kg rFVIIa was administered 3 h postextraction in 2021. The sinus membrane was exposed to the extraction socket, increasing the risk of epistaxis. He was discharged on POD 5 and POD 7 in 2019 and 2021, respectively, with no bleeding or thrombotic complications observed. Routine blood tests including complete blood count, prothrombin time, activated partial thromboplastin time, and fibrinogen were performed preoperatively and postoperatively. He was given oral tranexamic acid at 20–25 mg/kg every 8 h, from 2 h preintervention up to 7 days postintervention for all extractions except for the 2011 extraction where aPCC was administered. The introduction of emicizumab significantly reduced the hospitalization period from 8 to 3 days and markedly decreased the use of bypassing agents (BPAs).

## 3. Discussion

This study provides an intraindividual comparison of the perioperative management of tooth extractions in a PwHA-I before and after the initiation of emicizumab treatment. The results show that emicizumab reduces the use of BPAs, prevents postoperative bleeding, and shortens hospitalization. From an anatomical perspective, upper premolar and molar extractions may involve exposure or injury to the maxillary sinus membrane, leading to an increased risk of postoperative bleeding. In contrast, lower premolar and molar extractions pose a risk of damaging the inferior alveolar canal or its branches, which can result in significant hemorrhage. Furthermore, from a surgical standpoint, procedures requiring extensive bone removal are associated with higher bleeding risks. Such invasive procedures are more commonly performed in lower third molar extractions, where surgical difficulty tends to be greater. Therefore, taking both anatomical and procedural factors into account, it is generally considered that lower premolar and molar extractions have a higher risk of bleeding compared to their upper counterparts [15]. Nevertheless, in our reported case, no obvious difference in bleeding risk attributable to the extraction site was observed.

Moreover, while emicizumab has been shown to be generally safe, caution is necessary when it is used concomitantly with BPAs. The concomitant use of aPCC and emicizumab is discouraged due to the risk of thrombotic microangiopathy and thromboembolic events, especially at high cumulative doses.

Recent studies have reported successful procedures without additional use of factor infusions in patients on emicizumab [4, 5]. While this approach simplifies perioperative management and reduces costs, it may increase the risk of bleeding, particularly in more invasive procedures. Thus, careful individual risk assessment remains essential. The appropriate use of factor infusions during surgery offers the advantage of reducing the risk of postoperative bleeding.

However, it also carries the potential disadvantage of increasing the risk of thrombotic adverse events. Regardless of whether emicizumab prophylaxis is present, postoperative bleeding in haemophilia patients can result not only in direct physical harm but also in secondary burdens such as elevated healthcare costs and prolonged treatment periods. Therefore, it is important to thoroughly assess both local and systemic bleeding risks, and when the anticipated benefits outweigh the potential risks, preoperative factor infusion should be considered to optimize patient outcomes.

While emicizumab has ongoing costs, these may be offset by reduced hospitalization and BPA usage during surgeries [16, 17]. The absence of thrombotic complications further supports its safety.

## 4. Conclusion

Emicizumab prophylaxis significantly improves perioperative management of dental surgeries in PwHA-I by reducing BPA usage, preventing postoperative bleeding, and shortening hospital stays. These findings support the broader adoption of emicizumab prophylaxis for surgical procedures in this patient population.

## Figures and Tables

**Table 1:** Tooth extractions before and after emicizumab prophylaxis in a patient with haemophilia A and inhibitors.

Year	2011	2018	2019	2021
Emicizumab prophylaxis	No	Yes	Yes	Yes
Location of tooth extraction	Upper right 2nd premolar and 1st molar	Lower left 2nd premolar	Lower left 1st molar	Upper right 2nd molar
Preoperative BPA infusion				
rFVIIa (*μ*g/kg)	—	80	80	80
aPCC (U/kg)	80	—	—	—
Postoperative BPA infusion				
rFVIIa (*μ*g/kg)	1080	—	—	80
aPCC (U/kg)	640	—	—	—
Total BPA infusion				
rFVIIa (*μ*g/kg)	1080	80	80	160
aPCC (U/kg)	720	—	—	—
Number of instances of postextraction bleeding	3	0	0	0
Hospitalization period	8	3	3	3

Abbreviations: aPCC, activated prothrombin complex concentrates; BPA, bypassing agent; rFVIIa, recombinant factor VIIa.

## Data Availability

Research data are not shared.

## References

[1] Rider R. M., Rangel A. G., Preciado R. M., Carmona M. V. B., Ruiz S. I., Guillen A. P. (2018). Dental Management of a Child With Incidentally Detected Hemophilia: Report of a Clinical Case. *Case Reports in Dentistry*.

[2] Witmer C., Young G. (2013). Factor VIII Inhibitors in Hemophilia A: Rationale and Latest Evidence. *Therapeutic Advances in Hematology*.

[3] Oldenburg J., Mahlangu J. N., Kim B. (2017). Emicizumab Prophylaxis in Hemophilia A With Inhibitors. *New England Journal of Medicine*.

[4] Yagyuu T., Furukawa S., Zaizen M. (2023). Peri-Operative Hemostatic Management of Tooth Extraction in Patients With Hemophilia A, With and Without Inhibitors, Receiving Emicizumab Prophylaxis. *Haemophilia*.

[5] Ebbert P. T., Xavier F., Seaman C. D., Ragni M. V. (2020). Emicizumab Prophylaxis in Patients With Haemophilia A With and Without Inhibitors. *Haemophilia*.

[6] Mahlangu J., Oldenburg J., Paz-Priel I. (2018). Emicizumab Prophylaxis in Patients Who Have Hemophilia A Without Inhibitors. *New England Journal of Medicine*.

[7] Nogami K., Shima M. (2023). Current and Future Therapies for Haemophilia–Beyond Factor Replacement Therapies. *British Journal of Haematology*.

[8] Kruse-Jarres R., Peyvandi F., Oldenburg J. (2022). Surgical Outcomes in People With Hemophilia A Taking Emicizumab Prophylaxis: Experience From the HAVEN 1-4 Studies. *Blood*.

[9] Lewandowska M., Randall N., Bakeer N. (2021). Management of People With Haemophilia A Undergoing Surgery While Receiving Emicizumab Prophylaxis: Real-World Experience From a Large Comprehensive Treatment Centre in the US. *Haemophilia*.

[10] Yagyuu T., Yata S., Imada M. (2021). Risk Factors for Post-Extraction Bleeding in Patients With Haemophilia: A Retrospective Cohort Study. *British Journal of Oral & Maxillofacial Surgery*.

[11] Yagyuu T., Kawakami M., Ueyama Y. (2017). Risks of Postextraction Bleeding After Receiving Direct Oral Anticoagulants or Warfarin: A Retrospective Cohort Study. *BMJ Open*.

[12] Ruangchainicom N., Mahardawi B., Sakdejayont W. (2021). Topical Hemostatic Agents From an Oral-Surgery Perspective. *Journal of Oral and Maxillofacial Surgery, Medicine, and Pathology*.

[13] Bajkin B., Dougall A. (2020). Current State of Play Regarding Dental Extractions in Patients With Haemophilia: Consensus or Evidence-Based Practice? A Review of the Literature. *Haemophilia*.

[14] Zanon E., Martinelli F., Bacci C., Zerbinati P., Girolami A. (2000). Proposal of a Standard Approach to Dental Extraction in Haemophilia Patients. A Case-Control Study With Good Results. *Haemophilia*.

[15] Sumanth K. N., Prashanti E., Aggarwal H. (2018). Interventions for Treating Post-Extraction Bleeding. *Cochrane Database of Systematic Reviews*.

[16] Cortesi P. A., Castaman G., Trifiro G. (2020). Cost-Effectiveness and Budget Impact of Emicizumab Prophylaxis in Haemophilia A Patients With Inhibitors. *Thrombosis and Haemostasis*.

[17] Krishnamoorthy Y., Govindan D., Kannan N., Majella M. G., Hariharan V. S., Valliappan V. (2024). Budget Impact and Cost-Utility Analysis of Prophylactic Emicizumab Versus On-Demand Bypassing Agents for Adolescent Severe Haemophilia A Patients With Inhibitors in India. *Heliyon*.

